# Nutritional and economic evaluation of fermented durian peel on laying duck performance and yolk omega profile

**DOI:** 10.5455/javar.2026.m1004

**Published:** 2026-03-05

**Authors:** Jamila Mustabi, Nuraini Nuraini, Ade Trisna, Rahmad Fani Ramadhan, Athhar Manabi Diansyah, Nurazizah Nurazizah, Langgeng Priyanto, Herdis Herdis

**Affiliations:** 1 Department of Nutrition and Feed Technology, Faculty of Animal Science, Hasanuddin University, Makassar, Indonesia; 2 Department of Nutrition and Feed Technology, Faculty of Animal Science, Universitas Andalas, Padang, Indonesia; 3 Animal Science Department, Faculty of Agriculture, Universitas Sumatera Utara, Medan, Indonesia; 4 Department of Animal Nutrition and Feed Technology, Faculty of Animal Husbandry, Universitas Padjajaran, Bandung, Indonesia; 5 Department of Animal Production, Faculty of Animal Science, Hasanuddin University, Makassar 90245, Indonesia; 6 Animal Feed Technology Study Program, Faculty of Vocational, Hasanuddin University, Makassar, Indonesia; 7 Research Center for Animal Husbandry, National Research and Innovation Agency, Jl. Raya Bogor Km. 46, Cibinong, West Java 16911, Indonesia

**Keywords:** Fermented durian peel, *Lentinus edodes*, laying duck, yolk omega fatty acids, feed efficiency, IOFC

## Abstract

**Objectives:**

This study evaluated the effects of graded levels of Lentinus edodes–fermented durian peel (FDP) as a circular feed ingredient on production performance, yolk omega fatty acid composition, and economic efficiency of laying ducks, with the aim of identifying the optimal dietary inclusion level.

**Materials and Methods:**

A 12-week feeding trial was conducted using a completely randomized design with five dietary treatments containing 0, 6, 12, 18, and 24% FDP. Each treatment had four replicates with ten laying ducks per pen. Parameters measured included feed intake, feed conversion ratio (FCR), hen-day production (HDP), total egg production, egg weight, yolk omega-3, omega-6, and omega-9 fatty acids (analyzed by GC-FID), and income over feed cost (IOFC). Data was analyzed using ANOVA and correlation analysis.

**Results:**

Feed intake and egg weight differed significantly among treatments (*p* < 0.05), with reduced intake observed at the highest FDP inclusion level (24%). FCR, HDP, and total egg production were not significantly affected (*p* > 0.05). Yolk omega-6 fatty acid content increased significantly with FDP inclusion (*p* < 0.05), omega-3 showed selective treatment differences, and omega-9 remained unchanged. Correlation analysis indicated positive associations of omega-6 and omega-9 with feed intake and negative correlations with FCR, while omega-3 was positively correlated with feed intake. IOFC was highest at 12% FDP inclusion but declined to 24% due to reduced feed intake and egg weight.

**Conclusions:**

Fermented durian peel can be safely incorporated into laying duck diets at moderate levels, with 12% FDP identified as the optimal inclusion rate. At this level, production efficiency is maintained, yolk lipid quality is enhanced, and economic returns are maximized, while higher inclusion levels may negatively affect profitability.

## 1. Introduction

Feed costs are the primary determinant of duck egg production economics, driving ongoing efforts to identify locally available ingredients that can stabilize ration prices without compromising performance or product quality [1]. In Southeast Asia, durian (*Durio* spp.) processing generates substantial peel by-products that are often discarded, despite their carbohydrate-rich matrix and constituents suitable for valorization within circular bioeconomy frameworks [2]. However, the direct use of raw durian peel in monogastric diets is constrained by its high levels of structural fiber and lignin, which limit digestibility and voluntary intake [3]. Consequently, solid-state fermentation (SSF) has emerged as a scalable bioprocessing approach to enhance lignocellulosic residues by depolymerizing cell wall polysaccharides, reducing antinutritional factors, and increasing the availability of nutrients and small metabolites [4]. Among edible white-rot fungi, Lentinus edodes produces a complementary suite of cellulases, hemicellulases, and laccases that restructure fiber architecture under mild conditions, an enzymatic profile consistently linked to improved feed value of fibrous substrates for poultry [5].

In addition, SSF can modify phenolic profiles and release low-molecular-weight compounds that may affect gut physiology and lipid metabolism, suggesting that fermented ingredients may act as functional feed modifiers rather than inert diluents [6]. Suggest that converting durian peel through SSF into a feed ingredient could both mitigate waste and enhance the functional nutritional value of duck diets, warranting targeted *in vivo* evaluation. Durian peel exhibits a lignocellulosic matrix with cellulose around 57–64%, hemicellulose near 31%, and lignin around 13%, with reports of about 12.11% lignin in the rind [4]. These values explain the low digestibility of the unprocessed material in poultry, where lignin is essentially indigestible. Solid-state fermentation with edible white-rot fungi, such as Lentinula edodes, can confer ligninolytic and cellulolytic activities that remodel the cell wall network and reduce recalcitrant fiber fractions [3]. Studies on fermented durian waste for poultry indicate reduced crude fiber and a concurrent rise in crude protein after fermentation, consistent with enzyme-driven modification of the matrix [6].

In this study, we therefore quantify fiber fractions before and after fermentation and report percentage changes to evaluate process effectiveness. Indonesia’s laying duck systems are highly exposed to price volatility in maize, soybean meal, and fish meal markets, creating strong incentives to partially replace these conventional ingredients with nutritionally upgraded, locally sourced co-products [7]. Previous studies on the use of fermented agro-residues in poultry diets indicate that, when nutrient requirements are met, moderate inclusion levels can sustain feed intake, maintain laying performance, and prevent penalties in feed conversion ratio (FCR). However, responses vary depending on the ingredient, fermentation process, and poultry species, underscoring the need for duck-specific validation [8]. Robust evaluations in laying birds, therefore, emphasize pen-level feed intake measured alongside output periods, daily egg collection for egg number determination, and standardized assessment of egg weight, with FCR calculated over matched time intervals to accurately reflect efficiency [9].

Accordingly, a duck-focused evaluation of fermented durian peel, with precise intake and FCR measurements, is directly relevant to reducing adoption risks at the farm level. Attention has increasingly focused on yolk lipid quality. Dietary manipulation can alter the relative proportions of omega-3 (n-3), omega-6 (n-6), and omega-9 (n-9) fatty acids, with direct implications for consumer nutrition. Matrix effects arising from fermented botanicals, such as cell wall loosening, viscosity alterations, and phenolic lipid interactions, may further influence lipid digestion, micellization, and hepatic deposition, even when the overall fatty acid supply remains ostensibly unchanged [10, 11]. Studies involving fermented plant materials have associated these matrix alterations with changes in surrogate indicators of desaturase and elongase activity, as well as with selective partitioning within the n-3 and n-6 series. However, both the direction and magnitude of these effects vary depending on the substrate, microbial strain, inclusion level, and background diet [10]. Whether Lentinus edodes fermented durian peel (FDP) exerts neutral, beneficial, or adverse modulation of duck yolk fatty acids, therefore, remains an important question for value-added egg production [12].

Clarifying this response would support either the nutritional positioning of FDP-enriched eggs as sources of improved lipid profiles or the establishment of safe inclusion thresholds in the event of unfavorable shifts. Accordingly, we posit that the effects of fermented durian peel on yolk omega classes arise from matrix-driven shifts in lipid absorption, contributions of linoleic acid from fungal biomass, and phenolic-mediated modulation of hepatic desaturation rather than from gross fat supply. Any proposed feed ingredient must also meet a bioeconomic viability threshold. Income over feed cost (IOFC) serves as a concise decision metric that integrates ration cost, realized intake, laying performance, and prevailing market prices into a single profitability indicator [2]. Because FDP simultaneously modifies diet cost structure and may influence intake behavior, egg production, egg weight, FCR, and yolk composition, IOFC benchmarking across graded inclusion levels provides a practical bridge between controlled trials and farm decision-making. For ducks, however, the acceptable range of FDP inclusion in nutritionally balanced rations remains undefined. Dose-response effects on intake and FCR are still uncertain, and concurrent assessments of yolk omega profiles alongside IOFC under local price conditions are rarely reported [8, 13]. Demonstrating IOFC-neutral or IOFC-superior inclusion ranges under realistic economic scenarios would therefore provide actionable thresholds for both producers and integrators. The present study addresses these gaps by evaluating the graded inclusion of FDP (0, 6, 12, 18, and 24% of the diet) in nutritionally balanced rations and assessing its effects on laying performance (egg production and egg weight), intake and efficiency parameters (feed intake, FCR, and hen day egg production), yolk omega fatty acid profiles, and overall profitability under prevailing farm gate prices [9, 11]. By integrating performance, efficiency, lipid quality, and economic outcomes within a single analytical framework, this study aims to establish practical inclusion guidelines for FDP and contribute to the development of scalable circular feed strategies for Indonesia’s duck egg industry.

## 2. Materials and Methods

### 2.1. Ethical approval

All experimental procedures involving animals were reviewed and approved by the Animal Ethics Committee of the Faculty of Animal Science, Hasanuddin University, Makassar, Indonesia (Approval No.: 03976/UN4.1/KEP/2024). All husbandry practices and sample collections adhered to the national guidelines for the care and use of experimental animals. Every effort was made to minimize animal discomfort and to ensure their welfare throughout the study.

### 2.2. Experimental design

The study was conducted at the Faculty of Animal Science, Universitas Hasanuddin, Makassar, Indonesia. A completely randomized design (CRD) was employed with five dietary treatments based on fermented durian peel (FDP) inclusion: 0, 6, 12, 18, and 24% of the diet (T0–T4). A total of 200 laying ducks were randomly assigned to the five treatments, with four replicate pens per treatment and 10 ducks per pen (pen = experimental unit). The feeding trial lasted 12 weeks, following a brief acclimation period. Ducks were housed under standard management conditions, with ad libitum access to water. Feed was provided according to the experimental rations, and refusals were recorded to calculate feed intake. Health and welfare were monitored daily, and predefined humane endpoints were applied as necessary.

### 2.3. Fermentation of durian peel (FDP) and diet formulation

Fresh durian peels were trimmed to remove residual pulp, washed, and cut into 2–3 cm pieces. Substrate moisture was adjusted to 62–65% (wet basis), then autoclaved at 121°C for 20 min and cooled to ≤ 30°C [14]. A grain spawn of Lentinula edodes was added at 7% (w/w, wet basis) and mixed aseptically to ensure uniform distribution. Approximately 4.0 kg of inoculated substrate was loaded onto stainless trays at 3.5 kg of dry matter per tray (bed depth 3 cm). Fermentation proceeded for 10 days under aerobic static conditions at 26 ± 1°C with chamber relative humidity maintained at 85–90%; air exchange was passive via perforated lids and daily venting [15]. Trays were not turned. Endpoint criteria were ≥ 85% surface mycelial coverage with a stable tray-core temperature and pH 5.5–6.0. The fermented material was then dried at 55°C to ≤ 10% moisture and milled through a 1.0 mm screen to obtain FDP for diet formulation. Five iso-energetic, requirement-balanced diets containing FDP at 0, 6, 12, 18, and 24% (T0–T4) were prepared by proportional adjustment of basal ingredients to meet laying-duck requirements (Tables 1 and 2).

**Table 1.** Ingredient composition of experimental diets (% as-fed).

Ingredient	T0	T1	T2	T3	T4
Ground corn	55.00	50.00	48.00	49.50	42.00
Soybean meal	14.00	16.00	12.00	10.00	13.50
Fish meal	14.00	15.50	12.00	10.00	14.50
Fermented durian peel	0.00	6.00	12.00	18.00	24.00
Rice bran	15.00	10.00	13.50	10.00	3.00
Coconut oil	1.50	2.00	1.00	2.00	2.50
Top mix (vitamin–mineral premix)	0.50	0.50	1.00	0.50	0.50

**Table 2.** Analyzed nutritional composition of experimental diets (% dry basis).

Ingredient	T0	T1	T2	T3	T4
Crude protein (CP; %)	19.69	21.17	19.06	17.98	21.01
Ether extract (EE; %)	4.47	5.03	4.31	5.42	5.93
Crude fiber (CF; %)	5.18	5.21	6.20	6.43	6.24
Calcium (Ca; %)	3.20	3.47	3.01	2.36	3.22
Phosphorus (P; %)	0.79	0.80	0.91	0.71	0.75
Metabolizable energy (ME; kcal/kg)	2880	2901	2770.85	2898	2913

### 2.4. Production performance and efficiency

Production responses were monitored throughout the 12-week feeding period, with daily egg collection from each pen. The cumulative egg count over the observation period was recorded as egg production, and eggs collected on designated weighing days were individually weighed to calculate pen-level mean egg weight (gm) [16]. Feed intake was determined by recording feed offered and refusals on a routine schedule, with weekly verifications of totals [17]. Feed intake was calculated as the difference between feed offered and refusals and expressed as gm/duck/day after normalization by the number of ducks and number of days. Feed conversion ratio (FCR) was calculated at the pen level as feed intake (kg) divided by egg weight (kg) to maintain internal consistency between intake and output metrics [18]. Hen-day egg production (HDP, %) was calculated as [(eggs laid per day ÷ number of ducks) × 100] and averaged across the feeding period to obtain a pen-level value.

### 2.5. Egg yolk omega fatty acid profile

Eggs were collected daily and stored at ≤ 4°C. Yolks were separated, pooled by pen, homogenized for at least 30 sec, and aliquoted [19]. Total lipids were extracted using the Folch method (Chloroform: Methanol 2:1 v/v, with 0.01% BHT). The organic phase was washed with 0.88% KCl, dried over anhydrous, and the solvents were evaporated under at ≤ 35°C. Fatty-acid methyl esters (FAMEs) were prepared according to AOAC 996.06/ISO 5509, using base-catalyzed methanolic KOH, followed by acid methylation. FAMEs were extracted into n-hexane, washed with saturated NaCl, dried over, and brought to volume. Separation was performed by GC-FID on a polar cyanopropyl column (e.g., SP-2560; 100 m × 0.25 mm × 0.20 µm). Injector and detector temperatures were 260°C and 270°C, respectively; helium flow ≈ 1.0 ml/min; split ratio 1:50; injection volume 1 µl. Oven program: 140°C (5 min) → +4°C to 240°C; hold 15 min (lab-validated equivalents acceptable). Peaks were identified against authenticated FAME standards, and quantification was performed using methyl nonadecanoate (C19:0) as an internal standard. Each extract was injected in duplicate, with batch QA/QC including blanks and yolk reference material (retention time tolerance ± 0.03 min) [20].

### 2.6. Economic outcomes

Economic outcomes were evaluated using the income over feed cost (IOFC) ratio. The IOFC for each treatment was calculated as the difference between the revenue from egg sales and the corresponding feed expenditure over the observation period. Egg revenue was determined by the cumulative number of eggs produced per pen by the prevailing farm-gate price per egg at the study site. Feed expenditure was calculated based on the total feed consumed per pen and the unit cost of each diet, which reflected the actual ingredient prices for each formulation, including fermented durian peel (FDP). For ease of comparison, IOFC values were also expressed relative to the control group (T0 = 100%) to illustrate the proportional change across the graded FDP inclusion levels [2].

### 2.7. Statistical analysis

The experiment followed a completely randomized design (CRD) with the pen as the experimental unit. The effects of FDP inclusion levels (0, 6, 12, 18, and 24%) on feed intake, feed conversion ratio (FCR), hen day egg production (HDP), total egg production, egg weight, and yolk omega fatty acid composition were analyzed using one-way ANOVA. Results are presented as mean ± standard deviation (SD). Normality and homogeneity of variances were verified using the Shapiro–Wilk and Levene’s tests, respectively. When the overall F-test was significant (*α* = 0.05), pairwise comparisons among treatment means were conducted using Tukey’s honestly significant difference (HSD) test, and results were summarized using compact letter displays. Pearson correlation coefficients were calculated to examine relationships between production and efficiency variables and yolk omega fatty acid classes. Income over feed cost (IOFC) data were analyzed descriptively.

## 3. Results

### 3.1. Production performance and efficiency

**Figure 1.**
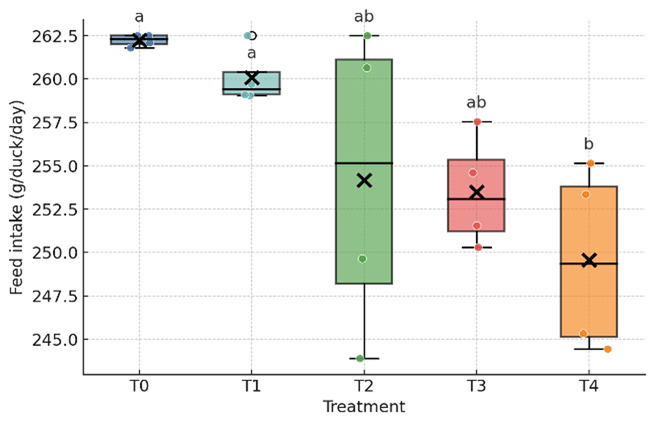
Feed intake of laying ducks fed diets containing graded levels of fermented durian peel (FDP). Different superscripts within a row indicate significant differences among treatments (*p* < 0.05). T0–T4 represent FDP inclusion levels of 0%, 6%, 12%, 18%, and 24% in the diet.

**Figure 2.**
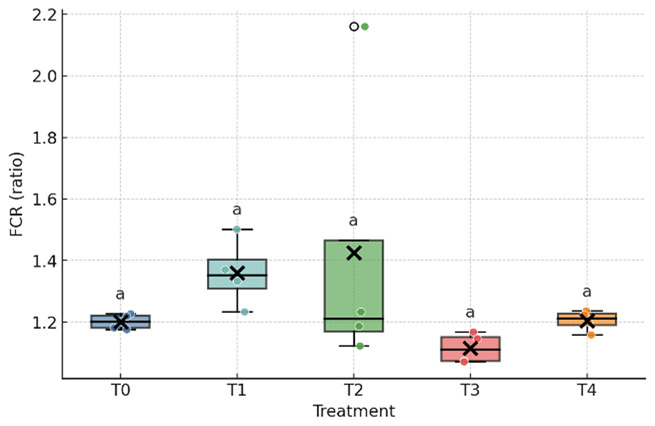
Feed conversion ratio (FCR) of laying ducks fed diets containing graded levels of fermented durian peel (FDP). Different superscripts within a row indicate significant differences among treatments (*p* < 0.05). T0–T4 represent FDP inclusion levels of 0%, 6%, 12%, 18%, and 24% in the diet.

**Figure 3.**
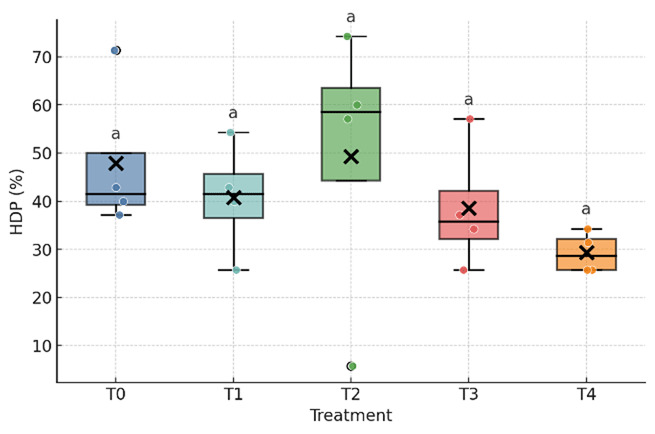
Hen-day production (HDP) of laying ducks fed diets containing graded levels of fermented durian peel (FDP). Different superscripts within a row indicate significant differences among treatments (*p* < 0.05). T0–T4 represent FDP inclusion levels of 0%, 6%, 12%, 18%, and 24% in the diet.

As shown in Table 3 and Figures 1, 2, 3, the inclusion of fermented durian peel (FDP) significantly affected feed intake and egg weight, whereas feed conversion ratio (FCR), hen day egg production (HDP), and total egg production remained unchanged. Feed intake decreased at the highest inclusion level (24%), with the T4 group differing significantly from T0 and T1, while T2 and T3 showed intermediate values that did not differ from either group. Egg weight was reduced at the 6% and 12% inclusion levels (T1 and T2) compared with T0 and T3, whereas T4 was intermediate and did not differ statistically from either group. No significant differences were observed among treatments for egg production, FCR, or HDP. Overall, FDP inclusion altered feed intake only at 24% and reduced egg weight at moderate levels (6–12%), without measurable effects on laying rate or feed efficiency during the study period.

**Table 3.** Egg production and egg weight of laying ducks fed diets containing graded levels of fermented durian peel (FDP).

Variable	Mean ± SD				
	T0	T1	T2	T3	T4
Egg production	16.75 ± 5,56^a^	14.25 ± 4.11^a^	17.25 ± 10.50^a^	13.50 ± 4.65^a^	10.25 ± 1.50^a^
Egg weight (gm)	62.35 ± 1.27^a^	54.90 ± 4.22^b^	54.86 ± 5.33^b^	65.04 ± 3.33^a^	59.19 ± 1.46^ab^

Note: Different superscripts within a row indicate significant differences among treatments (*p* < 0.05). T0–T4 represent FDP inclusion levels of 0%, 6%.,12%, 18%, and 24% in the diet.

### 3.2. Functional yolk omega fatty acid composition of eggs

As shown in Table 4, dietary inclusion of fermented durian peel (FDP) significantly affected yolk omega-6 fatty acids (*p* < 0.05), with compact letters indicating that T0 < T1 < T2, T3, and T4. Yolk omega-3 fatty acids also differed among treatments (*p* < 0.05); the letter groupings showed T0 a, T3 b, and T1, T2, and T4 ab, indicating at least one significant pairwise contrast, whereas other comparisons were not significant. In contrast, the omega-9 fraction did not differ among treatments (*p* > 0.05). Overall, FDP inclusion altered yolk omega-6 and omega-3 fatty acids but had no effect on omega-9 fatty acids.

**Table 4.** Yolk omega fatty acid composition of eggs from laying duck fed diets containing graded levels of fermented durian peel (FDP).

Variable	Mean ± SD				
**T0**	**T1**	**T2**	**T3**	**T4**
Omega-3 (%TFA)	0.09 ± 0.02^a^	0.07 ± 0.02^ab^	0.08 ± 0.02^ab^	0.06 ± 0.02^b^	0.08 ± 0.02^ab^
Omega-6 (%TFA)	7.58 ± 0.73^a^	9.37 ± 0.65^b^	10.12 ± 0.67^c^	10.22 ± 0.39^c^	10.49 ± 0.58^c^
Omega-9 (%TFA)	56.79 ± 6.62^a^	53.73 ± 6.44^a^	53.07 ± 7.32^a^	57.60 ± 3.77^a^	55.43 ± 3.04^a^

Note: Different superscripts within a row indicate significant differences among treatments (*p* < 0.05). T0–T4 represent FDP inclusion levels of 0%, 6%, 12%, 18%, and 24% in the diet.

### 3.3. Correlating yolk omegas with production and feed efficiency

**Figure 4.**
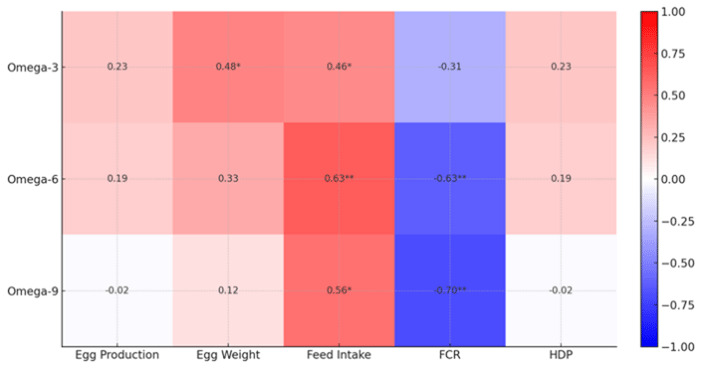
Pearson correlations between production and efficiency variables and yolk omega fatty acids in laying ducks fed diets containing graded levels of fermented durian peel (FDP). Blue indicates negative correlations, and red indicates positive correlations. Significance: *p* < 0.05 (*), *p* < 0.01 (**).

As shown in Figure 4, Pearson correlation analysis revealed selective associations between yolk fatty acid profiles and production or efficiency variables. Omega-3 fatty acids were positively correlated with egg weight and feed intake (*p* < 0.05) but showed no association with egg production, feed conversion ratio (FCR), or hen-day egg production (HDP) (*p* > 0.05). Omega-6 fatty acids were positively correlated with feed intake and negatively correlated with FCR (*p* < 0.01), with no significant relationships to egg production, egg weight, or HDP (*p* > 0.05). Similarly, omega-9 fatty acids showed positive correlations with feed intake (*p* < 0.05) and negative correlations with FCR (*p* < 0.01), while correlations with egg production, egg weight, and HDP were not significant (*p* > 0.05). Overall, higher feed intake was associated with greater yolk omega-3, omega-6, and omega-9 levels, whereas improved feed efficiency (lower FCR) was linked to higher omega-6 and omega-9 concentrations.

### 3.4. Economic outcomes

**Figure 5.**
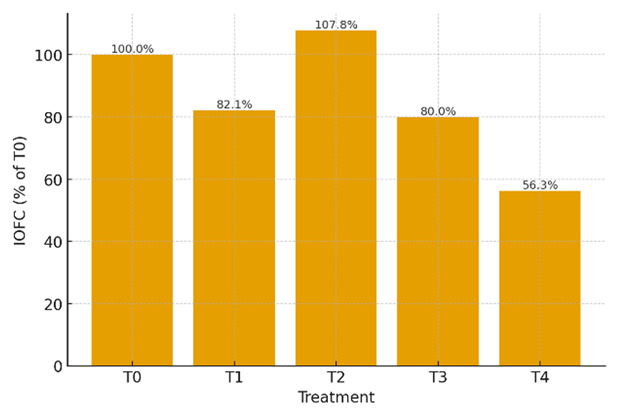
Income over feed cost (IOFC) of laying ducks fed diets containing graded levels of fermented durian peel (FDP).

As shown in Figure 5, income over feed cost (IOFC) varied across the different levels of fermented durian peel (FDP) inclusion. Relative to the control (T0), the 12% inclusion level (T2) yielded the highest IOFC, exceeding 100% of the T0 value. Treatments T1 and T3 showed IOFC values slightly below the control, whereas T4 recorded the lowest IOFC among all treatments.

## 4. Discussion

Dietary responses observed in this study are consistent with established effects of solid-state fermented lignocellulosic ingredients on intake regulation and matrix handling in poultry. Fermentation disrupts the plant cell wall, reduces selected antinutritional factors, and generates small metabolites that can enhance feed acceptance at moderate inclusion levels, whereas residual fiber bulk or flavor characteristics may limit intake at higher inclusion levels [21, 22, 23]. The stability of the feed conversion ratio (FCR) despite shifts in feed intake (Figures 1, 2, 3) suggests a pre-absorptive mechanism, primarily influenced by palatability, gastric fill, and early satiety cues, rather than systemic reductions in conversion efficiency when diets remain requirement balanced [24].

In comparison with prior work, dietary responses in this trial are broadly consistent with reports that solid-state fermented lignocellulosic residues improve acceptability at moderate inclusion levels, while very high levels can limit intake due to physical bulk or flavor notes, with the feed conversion ratio remaining stable when the formulation meets requirements [3]. The selective reduction in egg weight without a fall in laying rate fits mechanisms in which fermentation alters digesta viscosity, emulsification, and micellar transfer, thereby influencing yolk deposition more than ovulatory rhythm [24]. Importantly, the lower calculated metabolizable energy in T2 (2,770.85 kcal/kg) provides a plausible confounder for its smaller eggs, aligning with the previously described energy–egg mass coupling. The hierarchy of yolk fatty acids we observed—greater responsiveness of omega 6, modest change in omega 3 without a dedicated source, and buffering of omega 9—matches deposition control in birds and matrix-driven effects after fermentation that can shift absorption and hepatic routing [24]. Finally, the income over feed cost peaks at an intermediate inclusion, mirrors prior work where ration cost relief is offset beyond a turning point by biological drag, a concave pattern we corroborate with orthogonal polynomial contrasts indicating an interior optimum [10].

Observed differences in egg weight (Table 3), in the absence of parallel changes in laying rate, are consistent with matrix-driven modulation of lipid transport to the ovary. Fermentation can alter digesta viscosity and emulsification, which, in turn, affect micellar transfer, enterocyte uptake, and hepatic packaging of very low-density lipoprotein yolk, thereby influencing yolk deposition per egg more readily than ovulatory rhythm in managed flocks [25, 26]. Between-diet variation in nutrient density (Table 2), notably the lower ME in T2 (2,770.85 kcal/kg), likely influenced egg weight independent of FDP per se; we therefore interpret FDP effects alongside this background variation. Mechanistically, matrix loosening after fermentation can increase lipid bioaccessibility and alter bile-micelle dynamics; fungal biomass contributes linoleic-rich lipids (n-6 precursor); and phenolic-derived effects may modulate hepatic desaturase/elongase activity, together offering routes for the observed shifts in yolk omega classes. The yolk n-6: n-3 ratio increased with FDP (T0 84.22; T1 133.86; T2 126.50; T3 170.33; T4 131.12), indicating that stand-alone FDP is not suited for omega-3 enrichment claims; pairing FDP with n-3 sources would be required for a lower ratio.

Yolk fatty acid patterns (Table 4) and the correlation structure (Figure 4) align with the established hierarchy of fatty acid deposition in birds. Omega-6 fatty acids are highly diet-responsive because poultry depends largely on exogenous supply, whereas omega-3 shifts are modest unless supported by concentrated sources. Omega-9 is buffered by endogenous synthesis and homeostatic redistribution [27, 28]. Matrix loosening following fermentation can enhance lipid bioaccessibility and influence bile acid dynamics, providing a mechanistic explanation for the positive associations of omega-6 and omega-9 with feed intake and their negative correlations with feed conversion ratio (FCR). The absence of correlations with hen day egg production (HDP) suggests that these effects operate primarily through intake and digestive handling rather than through ovulation control [29, 30].

The economic ranking shown in Figure 5 reflects the combined effects of ration cost, actual feed intake, and egg-derived revenue, as integrated in income over feed cost (IOFC). Fermented co-products commonly reduce unit ration costs up to a threshold, beyond which biological constraints offset the savings, resulting in a profitability peak at intermediate inclusion levels when efficiency indices are maintained [31, 32]. In the present study, the inclusion level that balanced cost reduction with preserved feed intake and egg weight outperformed the control in terms of IOFC, whereas the highest inclusion level underperformed because reduced intake and non-superior egg weight lowered revenue more than the decrease in costs offset the cost.

The study did not quantify apparent digestibility, digesta viscosity, bile acid pools, or hepatic desaturase and elongase activities; therefore, mechanistic inferences remain indirect. Diets were formulated to meet nutrient targets rather than being confirmed through complete fatty acid analyses for each treatment, which limits the ability to separate supply effects from matrix effects. Replication was sufficient for primary endpoints, but larger cohorts and multi-phase sampling across the laying period would improve precision. Additionally, sensory and shelf-life attributes of eggs were not assessed, although these factors may be important for downstream product positioning. We also did not include a full treatment-wise IOFC breakdown table (feed intake, feed cost, hen-day production, revenue, and net income); while Figure 5 summarizes IOFC across treatments, a tabular decomposition would further enhance transparency and can be provided upon reasonable request. In addition, we did not report a complete nutritional composition of the FDP lot (e.g., proximate composition, amino acids, fatty acids, minerals), which limits the attribution of production responses specifically to the ingredient rather than to background nutrient substitution.

Taken together with Tables 3 and 4 and Figures 1, 2, 3, 4, 5, the findings present a pragmatic perspective. Fermented durian peel (FDP) functions as a locally sourced circular feed ingredient that can be included at intermediate levels, where feed intake remains stable, egg weight is maintained, and profitability benefits from reduced ration costs. Exceeding this inclusion window challenges palatability and physical matrix constraints, leading to reduced intake and egg size, which, in turn, limit efficiency and lower income over feed cost (IOFC). For practical application, inclusion should be anchored around this intermediate range, with pen-level feed intake and egg weight monitored as simple guardrails. When nutritional enhancement is desired, FDP can be paired with targeted lipid sources to modify yolk fatty acid profiles while maintaining economic performance. For future studies, the nutrient composition of all treatments should be kept constant to avoid bias in the results, unless differences in nutrient composition are intentionally incorporated into the experimental treatments.

## 5. Conclusions

In this study, incorporation of fermented durian peel (FDP) into requirement-balanced diets for laying ducks-maintained core production efficiency, with feed conversion ratio (FCR) and hen day egg production (HDP) remaining unchanged. Biological sensitivities were primarily reflected in feed intake and egg weight: intake decreased only at the highest inclusion level, while egg weight varied at intermediate levels. Yolk lipid composition shifted mainly through increased omega-6 fatty acids; omega-3 showed limited treatment effects, and omega-9 remained unchanged. Correlation analysis indicated that omega-6 and omega-9 increased with greater feed intake and were associated with improved efficiency, as reflected by lower FCR. Economically, income over feed cost (IOFC) peaked at intermediate inclusion levels, where cost reduction coincided with maintained intake and egg weight. At the highest inclusion, profitability declined because reduced revenue outweighed feed cost savings. Therefore, FDP is recommended to be included at approximately 12% in laying duck diets to optimize economic returns without compromising core production performance.

## Data Availability

The data presented in this study are available from the corresponding author upon reasonable request.

## List of abbreviations

AOAC, Association of Official Analytical Chemists; BHT, Butylated hydroxytoluene; CRD, Completely randomized design; DM, Dry matter; FAMEs, Fatty acid methyl esters; FCR, Feed conversion ratio; FDP, Lentinus edodes–fermented durian peel; GC-FID, Gas chromatography–flame ionization detector; HDP, Hen-day egg production; IOFC, Income over feed cost; ME, Metabolizable energy; n-3, Omega-3 fatty acids; n-6, Omega-6 fatty acids; n-9, Omega-9 fatty acids; QA/QC, Quality assurance/quality control; SSF, Solid-state fermentation; gm, grams; sec, seconds.

## References

[1] Ahmad SN, Rohaeni ES, Lase JA, Misbah A, Wardi W, Pasaribu T (2024). Effect of using shrimp head flour and small sea fish on duck egg production and egg quality. AIP Conf Proc.

[2] Khaksar G, Kasemcholathan S, Sirikantaramas S (2024). Durian (*Durio zibethinus* L.): nutritional composition, pharmacological implications, value-added products, and omics-based investigations. Horticulturae.

[3] Malenica D, Kass M, Bhat R (2023). Sustainable management and valorization of agri-food industrial wastes and by-products as animal feed: for ruminants, non-ruminants and as poultry feed. Sustainability.

[4] Egbune E, Ezedom T, Odeghe O, Egbune O, Eraga L, Avwioroko O (2025). Sustainable bioprocessing: Solid-state fermentation and agricultural waste. Afr Res Rep.

[5] Ezekiel LT, Abimbola FA, Mobolaji AO, Olusegun AO, Oluwaseyi AM, Adetola OO (2025). Enzymatic fungal bio-degradation of agro-industrial by-products for sustainable poultry nutrition: A review. World Poult Sci J.

[6] Green S, Eyres GT, Agyei D, Kebede B (2024). Solid-state fermentation: Bioconversions and impacts on bioactive and nutritional compounds in oats. Compr Rev Food Sci Food Saf.

[7] Ahmad SN, Tresia GE, Rohaeni ES, Bakrie B, Firison J, Lase JA (2024). Effect of calcium mineral supplementation in diets containing fishery waste on egg production and hatching performance of Mojosari ducks. Braz J Biol.

[8] Guo W, Xu LN, Guo XJ, Wang W, Hao QH, Wang SY (2022). The impacts of fermented feed on laying performance, egg quality, immune function, intestinal morphology and microbiota of laying hens in the late laying cycle. Animal.

[9] Li Y, Ma R, Qi R, Li J, Liu W, Wan Y (2024). Novel insight into feed conversion ratio in laying hens and construction of its prediction model. Poult Sci.

[10] Mens AJW, van Krimpen MM, Kar SK, Guiscafre FJ, Sijtsma L (2022). Enriching table eggs with n-3 polyunsaturated fatty acids through dietary supplementation with the phototrophically grown green algae *Nannochloropsis limnetica*: effects of microalgae on nutrient retention, performance, egg characteristics and health parameters. Poult Sci.

[11] Sharifi SD, Sanij MA, Rouhanipour H, Farzanegan A (2024). Diet enrichment with omega-3 fatty acids: Effects on performance, egg quality, yolk fatty acid profile, oxidative stability, and immunity. Acta Agric Scand A Anim Sci.

[12] Spim SRV, Oliveira BGCC, Leite FG, Gerenutti M, Grotto D (2017). Effects of *Lentinula edodes* consumption on biochemical, hematologic and oxidative stress parameters in rats fed a high-fat diet. Eur J Nutr.

[13] Zhu X, Tao L, Liu H, Yang G (2023). Effects of fermented feed on growth performance, immune organ indices, serum biochemical parameters, cecal odorous compound production, and the microbiota community in broilers. Poult Sci.

[14] Tambun R, Haryanto B, Alexander V, Manurung DR, Ritonga AP (2024). Durian peel (*Durio zibethinus*) utilization as an adsorbent in the purification of acidified crude glycerol. S Afr J Chem Eng.

[15] Ay C, Ülger İ, Kaliber M, Hızlısoy H, Ayasan T (2023). Effects of Shiitake mushroom (*Lentinus edodes*) supplementation into quail diets on performance, blood serum parameters and intestine microbial populations Effects of Shiitake Mushroom (*Lentinus edodes*) Supplementations into Quail Diets. J Hellenic Vet Med Soc.

[16] Portillo-Salgado R, Cigarroa-Vázquez FA, Ruiz-Sesma B, Mendoza-Nazar P, Hernández-Marín A, Esponda-Hernández W (2021). Prediction of egg weight from external egg traits of guinea fowl using multiple linear regression and regression tree methods. Braz J Poult Sci.

[17] Nurlatifah A, Astuti DA, Herdis H, Arifiantini I, Pamungkas FA, Santoso S (2025). Mitigating heat stress in Garut lambs: Synergistic effects of Lemuru fish oil, vitamin E, and selenium on antioxidant defense, hematology, and physiological responses. Vet World.

[18] Davison C, Michie C, Tachtatzis C, Andonovic I, Bowen J, Duthie CA (2023). Feed conversion ratio (FCR) and performance group estimation based on predicted feed intake for the optimisation of beef production. Sensors.

[19] Rouhanipour H, Ashayerizadeh O, Sharifi SD, Dastar B (2025). Copper and L-arginine supplementation: impacts on laying hen health and productivity. Ital J Anim Sci.

[20] Ochoa-Herrera V, Quintanilla F, Egas DA, Mora JR (2021). Optimization of gas chromatography methodology for biodiesel analysis. J Anal Chem.

[21] Katu JK, Tóth T, Varga L (2025). Enhancing the nutritional quality of low-grade poultry feed ingredients through fermentation: A review. Agriculture.

[22] Obianwuna UE, Huang L, Zhang H, Wang J, Qi G, Qiu K (2024). Fermented soybean meal improved laying performance and egg quality of laying hens by modulating cecal microbiota, nutrient digestibility, intestinal health, antioxidant and immunological functions. Anim Nutr.

[23] Nurlatifah A, Prihantoko KD, Menassol JB, Herdis H, Diansyah AM, Khan FA (2025). Water hyacinth: A promising functional feed ingredient to optimize reproductive performance in Garut rams. Open Vet J.

[24] Clark CEF, Akter Y, Hungerford A, Thomson P, Islam MR, Groves PJ (2019). The intake pattern and feed preference of layer hens selected for high or low feed conversion ratio. PLoS One.

[25] Upadhaya SD, Lee JS, Jung KJ, Kim IH (2018). Influence of emulsifier blends having different hydrophilic-lipophilic balance value on growth performance, nutrient digestibility, serum lipid profiles, and meat quality of broilers. Poult Sci.

[26] Guo H, Zhang X, You M, Shen Y, Zhang S, Li J (2024). Quantitative lipidomics reveals the changes of lipids and antioxidant capacity in egg yolk from laying hens with fatty liver hemorrhagic syndrome. Poult Sci.

[27] Wang S, Mohammed KAF, Zhang Y, Ruan D, Xia W, Fouad AM (2021). Nutritional impacts of using graded levels of dietary linoleic acid on egg production, egg quality, and yolk fatty acid profile of laying ducks. Ital J Anim Sci.

[28] Lee SH, Kim YB, Kim DH, Lee DW, Lee HG, Jha R (2021). Dietary soluble flaxseed oils as a source of omega-3 polyunsaturated fatty acids for laying hens. Poult Sci.

[29] Arshad MA, Faiz-ul-Hassan, Bhatti SA, Rehman MSU, Yousaf W, Younus G (2021). Supplementation of bile acids and lipase in broiler diets for better nutrient utilization and performance: Potential effects and future implications – a review. Ann Anim Sci.

[30] Yin C, Xia B, Tang S, Cao A, Liu L, Zhong R (2021). The effect of exogenous bile acids on antioxidant status and gut microbiota in heat-stressed broiler chickens. Front Nutr.

[31] Al-Khalaifah HS, Shahin SE, Omar AE, Mohammed HA, Mahmoud HI, Ibrahim D (2020). Effects of graded levels of microbial fermented or enzymatically treated dried brewer’s grains on growth, digestive and nutrient transporter genes expression and cost effectiveness in broiler chickens. BMC Vet Res.

[32] Ibrahim D, El-Sayed HI, Mahmoud ER, Abdel-Rahman GIA, Bazeed SM, Abdelwarith AA (2023). Impacts of solid-state fermented barley with fibrolytic exogenous enzymes on feed utilization, and antioxidant status of broiler chickens. Vet Sci.

